# Mandatory vaccination in child daycare and its relevance to coronavirus disease 2019

**DOI:** 10.3325/cmj.2021.62.638

**Published:** 2021-12

**Authors:** André den Exter

**Affiliations:** Erasmus School of Law, Erasmus University Rotterdam, Rotterdam, the Netherlands

On April 8, 2021, the European Human Rights Court in Strasbourg made a landmark ruling on mandatory vaccination of children (*Vavricka v the Czech Republic*, no 47621/13, 8 April 2021) (1). After a long legal battle lasting 16 years, the Grand Chamber decided that a Czech national law imposing a statutory duty of a set of standard vaccinations for children under the age of 15 (2) does not violate the right to private life as protected under the European Convention on Human Rights (ECHR).

Although the outcome of this ruling is not surprising, it may also have consequences relating to the controversy of mandatory coronavirus disease 2019 (COVID-19) vaccination, which has been raised in other European countries.

According to the Czech Public Health Act, all permanent residents should be vaccinated for polio, hepatitis B, and tetanus. These vaccinations are mandatory for children being admitted to preschool facilities – although the domestic system does allow exemptions for specific medical reasons. Parents who fail to comply with this parental duty risk a penalty of around €400. However, the Czech system excludes forced vaccination.

Vavricka, and other parents, were found guilty of non-compliance to vaccinate their children and ordered to pay a penalty. They challenged the constitutionality of that decision, claiming that the vaccination duty violated their parental right to refuse medical treatment under the Oviedo Convention, as part of the Czech legal order. In the end, the Constitutional Court dismissed the complaint, arguing that in principle compulsory vaccination can be considered an admissible limitation of the individual’s human rights.

Ultimately, the case was submitted to the Strasbourg Human Rights Court, where Mr Vavricka complained about the arbitrary nature of the penalty and the failure to comply with the right to respect his private life as protected under the ECHR.

In the Court’s assessment, a person’s physical integrity is inherent to the private life concept, while compulsory vaccination, as an involuntary medical intervention, represents an interference with the right to respect for private life, even though the vaccination was not enforced.

Since the right to private life is not an absolute right, restrictions can be allowed when justified. But, under these circumstances, the restriction should comply with several conditions, including a proper legal basis approved by Parliament (namely, the Czech Public Health Act and Education Act). Moreover, the aim of the vaccination duty should be legitimate, in this instance protecting society at large against the contagious diseases in question. This corresponds with the protection of public health and the rights of others, which is recognized by the treaty right. And, finally, the interference should be “necessary in a democratic society” to “answer a pressing social need.”

In controversial cases, like most health care policy issues are, the Court leaves the countries a wide margin of appreciation, as they are the most competent to decide on these sensitive matters, as long as they comply with the treaty’s core principles. Thus, where there is no consensus about the system of vaccination – ie, whether it is voluntary or mandatory – it remains up to the member state to decide what is the most suitable approach to meet the social pressing need (ie, solving the health crisis), in the least restrictive manner, and balancing public and individual rights. Taking into account the decrease in voluntary vaccination in several member states, the duty to be vaccinated is not considered unreasonable but, rather, is viewed as an element of “social solidarity” to protect the health of others, particularly that of vulnerable groups. After all, the right to private life does not only include an obligation to abstain from unlawful interference in a person’s private life, but, simultaneously, includes a generally recognized positive obligation to protect the life and well-being of others from health risks. Under these circumstances, in this case the Court ruled by majority that the ECHR’s right to private life is not violated.

Although the case concerns contagious diseases other than COVID-19, the judgment might also legitimize mandatory vaccination measures in the case of the current pandemic, not restricted to preschool facilities. Mandatory vaccination, not forced vaccination, can therefore be justified as complying with a social pressing need. Even when there is no 100% guarantee of its effectiveness, and particularly when vaccine hesitancy is increasing.
